# The Local Treatment of Croup

**Published:** 1870-10

**Authors:** Adolphe Weber


### The Local Treatment of Croup.

BY ADOLPHE WEBER, M. D.

A knowledge of the power possessed by lactic acid to dissolve fibrinous exudations, induced the author to try it in cases of croup. At first he used it only after the operation of tracheotomy, partly with a view to keep the tracheotomy tubes clean, and partly hoping that the lactic acid might affect the membranes which extended downwards into the bronchi. The results were so favorable in both respects that he proceeded to try it in severe cases of croup before having recourse to tracheotomy. Since then he has not once had occasion to operate, and has not lost a single case of croup. In some very severe cases in which inspiration and expiration were equally obstructed, and the condition of the fauces indicated an abundant fibrinous exudation in the trachea, the difficulty of breathing was completely relieved within seven to ten hours of using this remedy, and two or three days after no trace of the local affection remained.

During the treatment there was not, as is generally the case, an expectoration of tough membranous sputa, but gradually the whistling, barking inspiration and expiration were replaced by distinct rattling noises; the voice before, quite suppressed, began to assume a hoarse timbre, and considerable quantities of loose white frothy phlegm were expectorated during the fits of coughing, until at last the struggle for breath quite ceased, and the disease assumed more the character of a catarrhal affection of the throat.

The treatment consists in the local application of the remedy to the windpipe by means of inhalation. The patient is made to inhale a solution of lactic acid (15 to 20 drops in half an ounce of water) at first every half-hour, and afterwards, when the respiration improves, every hour or every two hours a solution of 10 to 15 drops in half an ounce of water.

The inhalation is discontinued as soon as the dyspnœa has subsided, and to promote expectoration chamomile tea is exhibited.

In using the inhalation care must be taken that the vapor does not affect the eyes or face.

With this treatment was conjoined the internal exhibition of carbonate of soda every half-hour or every hour, which was thought to exert a beneficial effect upon the exudation.—*Medical Times and Gazette*, January 22.—(*Half-Yearly Abstract.*)

